# Stand Density Drives Soil Microbial Community Structure in Response to Nutrient Availability in *Larix gmelinii var. principis-rupprechtii* (Mayr) Pilger Plantations

**DOI:** 10.3390/plants14243737

**Published:** 2025-12-08

**Authors:** Fengzi Li, Lei Liu, Long Hai, Hongwei Yang, Kai Zhao, Qiuming Di, Zhibo Wang

**Affiliations:** 1 Inner Mongolia Academy of Forestry Sciences, Hohhot 010010, China; fengzili@emails.imau.edu.cn (F.L.);; 2 Inner Mongolia Daqingshan Forest Ecosystem Positioning Observation and Research Station, Wuchuan 011700, China; 3 College of Desert Control Science and Engineering, Inner Mongolia Agricultural University, Hohhot 010010, China; 4 College of Forestry, Inner Mongolia Agricultural University, Hohhot 010018, China

**Keywords:** *Larix gmelinii var. principis-rupprechtii* (Mayr) Pilger, plantation forest, plant–microbe–soil interactions, stand density, microbial diversity

## Abstract

Sustainable forest management requires a comprehensive understanding of how stand density regulates soil ecological processes. We examined a *Larix principis-rupprechtii* plantation under three thinning retention densities (High—HD; Medium—MD; Low—LD) and an unthinned control (CK), with soil samples collected from four depth layers (0–10, 10–20, 20–30, and 30–40 cm). This study investigated the effects of stand density on soil properties and microbial communities in a *Larix principis-rupprechtii* plantation by combining high-throughput sequencing with soil physicochemical analysis to identify the optimal density regime for maintaining soil health. Results demonstrated the following: (1) Moderate-density (MD) management best balanced the stability of soil ecosystem structure, showing superior water retention, organic carbon content, and microbial diversity in the 0–30 cm soil layer. The mechanism underlying these improvements can be attributed to the moderately open canopy structure in MD stands, which facilitated efficient litter decomposition and drove functional complementarity between *Basidiomycota* (enhancing cellulose degradation capacity) and *Acidobacteriota* (adapted to oligotrophic conditions). (2) Redundancy analysis revealed that soil pH and available nutrients (AK, AP) were key environmental factors driving microbial community restructuring: *Actinobacteriota* dominated in neutral, phosphorus-rich environments, while *Acidobacteriota* thrived under acidic, phosphorus-limited conditions. Fungal communities showed high sensitivity to management intensity, with significant shifts between *Ascomycota* and *Basidiomycota*, whereas bacterial communities remained relatively stable due to functional redundancy. We recommend the adoption of moderate-density management as a sustainable practice to enhance soil nutrient cycling and maintain microbial diversity, thereby providing scientific support for sustainable plantation management.

## 1. Introduction

Forest ecosystems are a vital component of the terrestrial biosphere, whose structure and function are influenced by a variety of biological and abiotic factors. Among these, stand density—a key indicator in forest management—directly affects the micro-environmental conditions within forests (such as light, temperature, and humidity) and soil physicochemical properties (such as organic matter content, pH, and nutrient cycling), thereby regulating belowground ecological processes [1]. Soil microorganisms, as core drivers of the forest belowground ecosystem, participate in critical ecological functions including organic matter decomposition, nutrient transformation, and plant–soil feedbacks [2]. Stand density indirectly alters soil microclimate and resource availability by regulating canopy structure and understory conditions. High stand density typically leads to reduced light penetration and increased humidity, which slows down litter decomposition and affects the input of soil organic matter. In contrast, low stand density, characterized by sufficient light and better ventilation, may accelerate litter mineralization and promote nutrient release [3]. Furthermore, stand density influences root distribution and the composition of root exudates, thereby modifying the availability of soil carbon sources [4]. These changes may further impact microbial metabolic activity and community structure.

Soil microorganisms (including bacteria, fungi, archaea, etc.) are key biological groups driving material cycling and energy flow in forest ecosystems. Bacteria (such as *Proteobacteria* and *Acidobacteria*) play important roles in carbon and nitrogen cycling, while fungi (such as *Ascomycota* and *Basidiomycota*) predominantly govern lignin degradation and mycorrhizal symbiosis [5]. Microbial diversity is generally positively correlated with ecosystem stability, where high diversity implies stronger functional redundancy and environmental adaptability [6]. Therefore, understanding how stand density affects microbial community composition and diversity contributes to evaluating the long-term impacts of different management models on soil health.

Currently, there is a growing body of research on the effects of stand density on soil microorganisms, yet the findings remain inconsistent. For example, some studies suggest that high stand density may promote the enrichment of certain functional microbial groups (such as saprotrophic fungi) due to increased litter input [7]; whereas others have found that low stand density, with its higher resource availability, may support greater microbial diversity [8]. These discrepancies may arise from confounding factors such as forest type, climatic conditions, or soil properties. Moreover, most existing studies focus solely on individual groups such as bacteria or fungi, overlooking the integrated response of the microbial community and its functional implications.

*Larix gmelinii var. principis-rupprechtii* (Mayr) Pilger *(Larix principis-rupprechtii)*, as a pioneer afforestation species in northern China, widely distributed across the North and Northwest regions, and is characterized by its strong adaptability and rapid growth. In recent years, due to the large-scale establishment of plantations, the impact of its management practices-such as thinning and density control-on ecosystem functioning has garnered increasing attention. However, research on how stand density affects soil microbial communities by altering the soil micro-environment remains limited, particularly in temperate plantation ecosystems. Therefore, this study investigates the influence of different stand densities on the composition and diversity of soil microorganisms in Chinese larch plantations, aiming to provide a scientific basis for optimizing forest management strategies and sustaining soil ecological functions.

## 2. Results

### 2.1. Differences in Soil Physicochemical Properties Among a Larix principis-rupprechtii Plantations with Different Stand Densities

#### 2.1.1. Differences in Soil Nutrient Content

**Figure 1:**
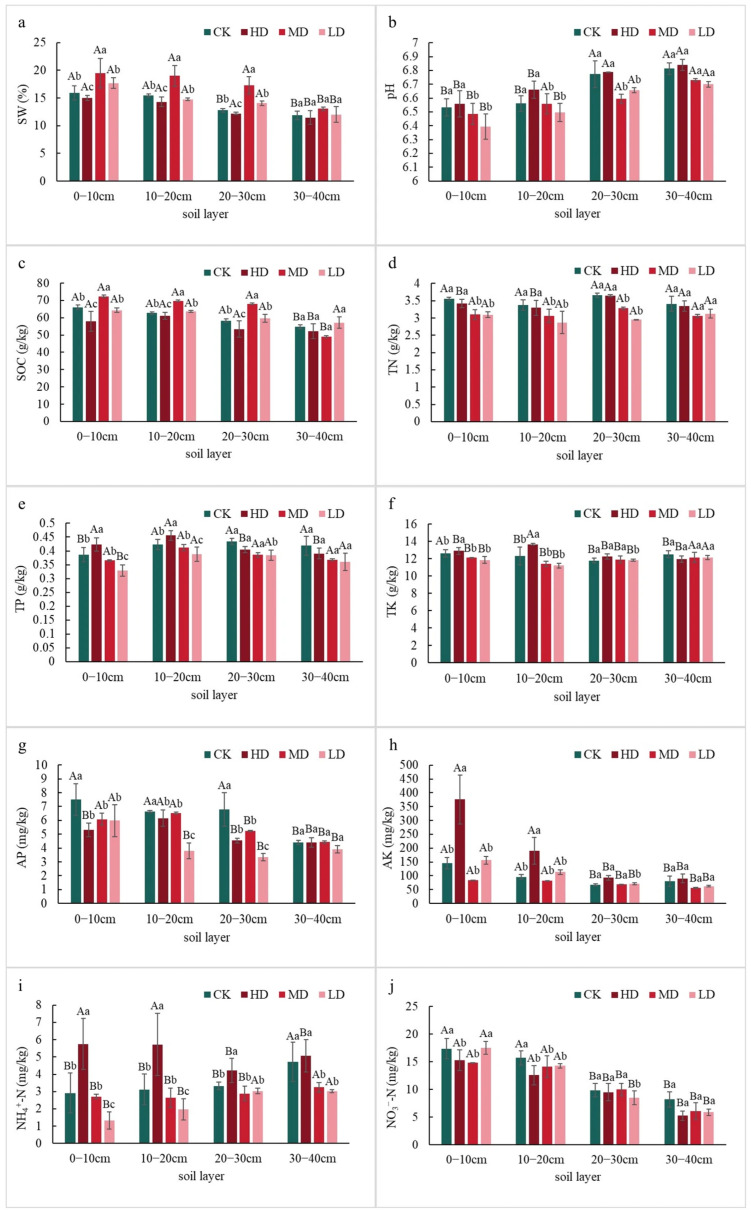
Effects of different stand retention densities on soil physicochemical properties in a *Larix principis-rupprechtii* plantation. **Note:** Different uppercase letters indicate significant differences among soil layers within the same stand density, and different lowercase letters indicate significant differences among stand densities within the same soil layer (*p* < 0.05).

Soil water content (SW), pH, soil organic carbon (SOC), total nitrogen (TN), total phosphorus (TP), total potassium (TK), available phosphorus (AP), available potassium (AK), ammonium nitrogen (NH_4_^+^-N), and nitrate nitrogen (NO_3_^−^-N) content were significantly influenced by different stand retention densities (Figure 1). These parameters generally decreased with increasing soil depth, with all treatments showing significantly higher values in the 0–20 cm layer than in the 20–40 cm layer (*p* < 0.05). Soil pH decreased significantly with decreasing stand density (*p* < 0.05), with all soil layers being weakly acidic (range: 6.38–6.89). In the 0–30 cm layer, SW and SOC initially increased and then decreased with reduced stand density, with the moderate density (MD) treatment being significantly higher than the high density (HD), low density (LD), and control (CK) treatments (*p* < 0.05). No significant differences were observed in the 30–40 cm layer (*p* > 0.05). In the 0–20 cm layer, TP and TK decreased with decreasing stand density, with HD being significantly higher than MD, LD, and CK (*p* < 0.05). No significant differences were found in the 20–40 cm layer (*p* > 0.05). AP in the 0–30 cm layer was lower than in CK, with LD being significantly lower than HD, MD, and CK (*p* < 0.05). No significant differences were detected in the 30–40 cm layer (*p* > 0.05). In the 0–20 cm layer, AK exhibited a “V”-shaped pattern with decreasing stand density, and HD was significantly higher than the other treatments (*p* < 0.05). No significant differences were observed in the 20–40 cm layer (*p* > 0.05). NH_4_^+^-N content decreased with decreasing stand density across all soil layers, and HD was significantly higher than the other treatments (*p* < 0.05). NO_3_^−^-N content in the 0–30 cm layer decreased with decreasing stand density (*p* < 0.05), while no significant differences were found in the 30–40 cm layer (*p* > 0.05).

#### 2.1.2. Differences in Soil Enzyme Activity

**Figure 2:**
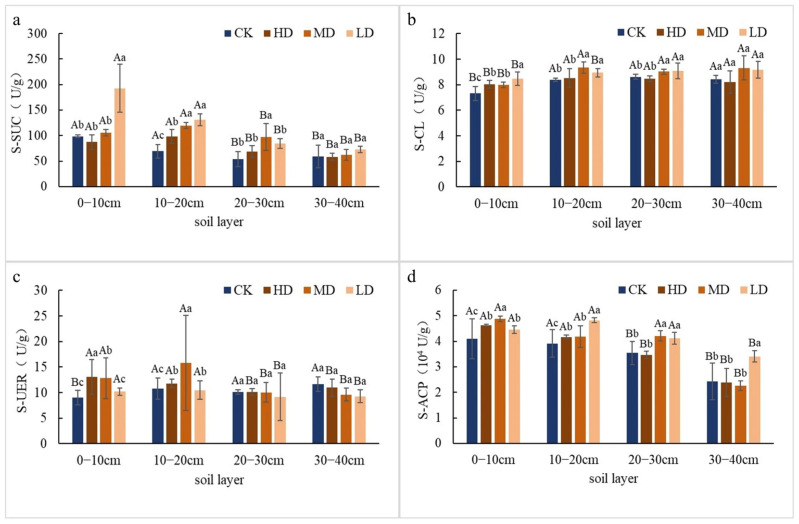
Effects of different stand retention densities on soil enzyme activities in a *Larix principis-rupprechtii* plantation. **Note:** Different uppercase letters indicate significant differences among soil layers within the same stand density, and different lowercase letters indicate significant differences among stand densities within the same soil layer (*p* < 0.05).

Different stand densities significantly affected the activities of soil sucrase (S-SUC), cellulase (S-CL), urease (S-URE), and acid phosphatase (S-ACP) (Figure 2). In the 0–30 cm layer, S-SUC and S-CL activities were decreased with reducing stand density, where it was significantly higher in LD and MD than in HD and CK (*p* < 0.05), with no significant differences in the 30–40 cm layer. S-URE activity was higher in HD and MD than in LD and CK in the 0–20 cm layer (*p* < 0.05), but showed no significant differences in the 20–40 cm layer. S-ACP activity decreased with decreasing stand density and was significantly higher in MD and LD than in HD and CK (*p* < 0.05).

### 2.2. Soil Microbial Community Structure in Larix principis-rupprechtii Plantations Under Different Stand Densities

#### 2.2.1. Micro Differences in the Species-Level Structure of Soil Microorganisms

**Figure 3:**
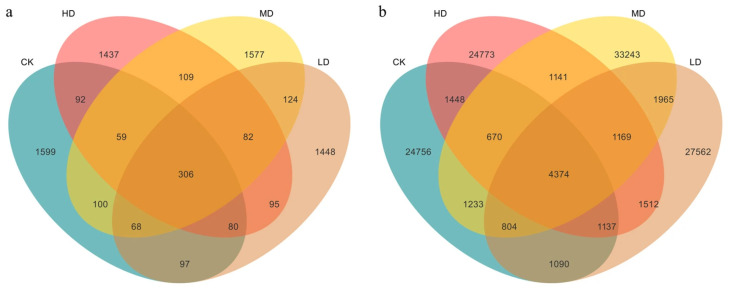
Differences in soil fungal (**a**) and bacterial (**b**) species composition in a *Larix principis-rupprechtii* plantation under different stand densities.

Venn diagrams illustrate the number of shared and unique ASVs across samples. Under the four stand density treatments, 306 fungal ASVs and 4374 bacterial ASVs were shared among all groups (Figure 3). For the fungal community (Figure 3a), the CK treatment contained the highest number of unique ASVs (1599), followed by MD (1577). As stand density increased, the number of unique fungal ASVs decreased, with the HD treatment showing the fewest (1437). In the bacterial community (Figure 3b), the MD treatment supported the most unique ASVs (33,243), followed by LD (27,562), while HD had the fewest (24,773). This indicates that moderate stand density favors the presence of unique bacterial taxa, whereas high stand density may negatively affect the diversity of certain bacterial species. Both bacterial and fungal ASV counts were lowest under the HD treatment, suggesting that excessively high stand density may suppress soil microbial diversity.

#### 2.2.2. Differences in the Phylum-Level Structure of Soil Microorganisms

**Figure 4:**
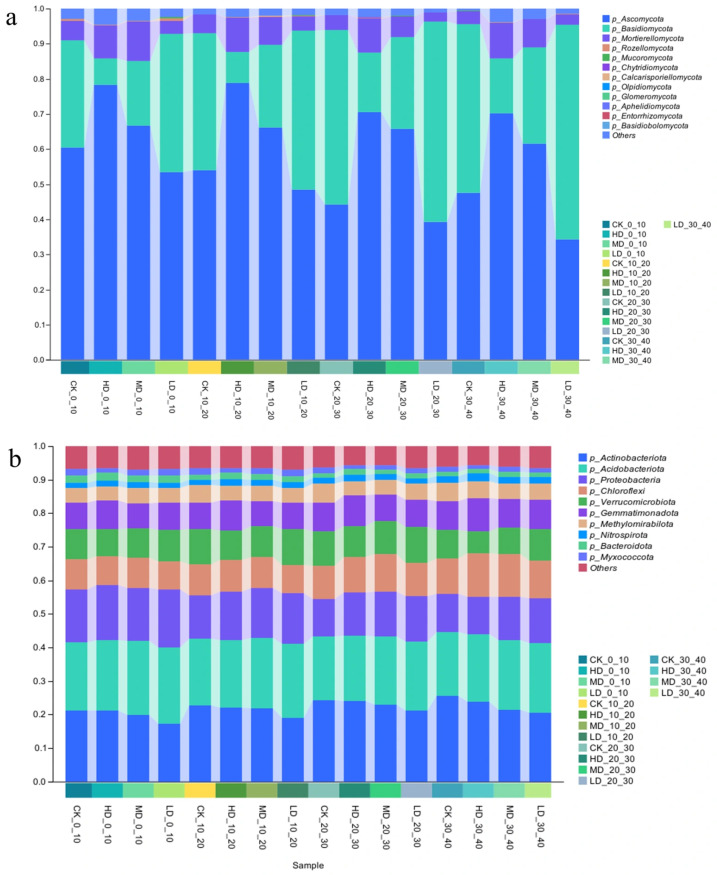
Impact of stand retention density on relative abundance of fungal (**a**) and bacterial (**b**) phyla in a *Larix principis-rupprechtii* plantation under different stand densities.

Figure 4 shows that 16 fungal (677 genera) and 43 bacterial phyla (887 genera) were identified in the *Larix principis-rupprechtii* plantation. The fungal community was dominated by *Ascomycota* (58.73% ± 15.27%), *Basidiomycota* (32.41% ± 18.55%), and *Mortierellomycota* (6.54% ± 3.31%), collectively representing 96.46–98.07% of total composition (Figure 4a). With decreasing stand density, *Ascomycota* and *Mortierellomycota* declined while *Basidiomycota* increased, indicating a shift toward *basidiomycete* dominance. The bacterial community was primarily composed of *Actinobacteriota* (21.81% ± 2.35%), *Acidobacteriota* (20.60% ± 1.46%), and *Proteobacteria* (13.92% ± 2.09%), totaling 55.86–56.83% (Figure 4b). Reduced stand density decreased *Actinobacteriota* but increased *Acidobacteriota* and *Proteobacteria*. Specifically, *Actinobacteriota* and *Acidobacteriota* abundances shifted from 22.89%/20.33% (high density) to 21.64%/21.25% (medium), and 19.69%/21.61% (low density). Under low density, *Acidobacteriota* surpassed *Actinobacteriota* as the most abundant phylum, indicating a density-dependent shift in bacterial dominance.

### 2.3. Soil Microbial Diversity Analysis in Larix principis-rupprechtii Plantations Under Different Stand Densities

#### 2.3.1. Alpha Diversity Analysis

**Figure 5:**
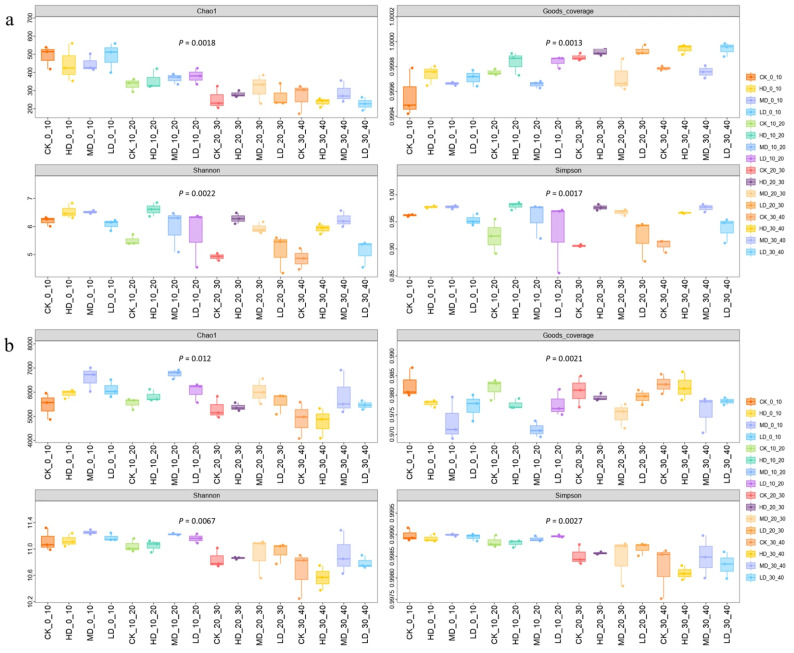
Effects of different stand retention densities on soil fungal (**a**) and bacterial (**b**) alpha diversity indices in a *Larix principis-rupprechtii* plantation.

For fungal diversity (Figure 5a), the Chao1 index decreased with soil depth. In the 0–20 cm layer, it increased with decreasing stand density, peaking in the LD treatment. In the 10–20 cm layer, it showed a hump-shaped trend, reaching its highest value under MD. These results suggest that low to moderate densities significantly enhance fungal richness. The coverage index approached 0.998 across samples, indicating high sequencing depth. It exhibited a “V”-shaped pattern with decreasing stand density, with higher values in the HD treatment. The Shannon and Simpson indices decreased with reduced density in the 0–30 cm layer (highest in HD), while in the 30–40 cm layer, they followed a hump-shaped pattern, peaking under MD.

For bacterial diversity (Figure 5b), the Chao1 index showed a hump-shaped response to decreasing stand density, with MD yielding the highest value, indicating enhanced richness at moderate density. The coverage index (≈0.980) confirmed sufficient sequencing depth and followed a trend similar to that of fungi. The Shannon index also exhibited a hump-shaped pattern, maximized under MD. In the 0–30 cm layer, the Simpson index was higher in MD and LD, suggesting dominant species exerted stronger influence under these treatments. In summary, stand density had a more pronounced impact on fungal diversity, with MD most effectively enhancing both fungal richness and diversity. Bacterial diversity was less affected by density variation, though MD still provided the most favorable conditions.

#### 2.3.2. Principal Coordinate Analysis

**Figure 6:**
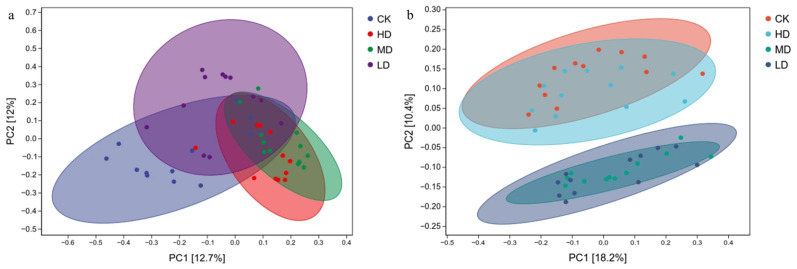
Principal coordinate analysis of soil fungal (**a**) and bacterial (**b**) communities under different stand retention densities in a *Larix principis-rupprechtii* plantation.

Principal coordinate analysis (PCoA) based on Bray–Curtis distances at the genus level revealed that for fungal communities, PC1 and PC2 explained 12.70% and 12.00% of the total variation, respectively (Figure 6). These components were identified as the main factors driving differences among samples, indicating that stand density significantly influenced soil fungal community structure. Fungal communities in HD and MD clustered closely, indicating minimal compositional differences between these densities (Figure 6a). In contrast, samples from other stand densities showed greater dispersion, demonstrating distinct structural variations. For bacterial communities (Figure 6b), PC1 and PC2 accounted for 18.20% and 10.40% of the variation, respectively. Bacterial communities in CK and HD clustered together, as did those in MD and LD, suggesting relatively similar compositions within these pairs. However, significant separation was observed between the CK/HD and MD/LD clusters, indicating substantial compositional differences among these treatment groups.

### 2.4. Correlations Among Soil Physicochemical Properties

**Figure 7:**
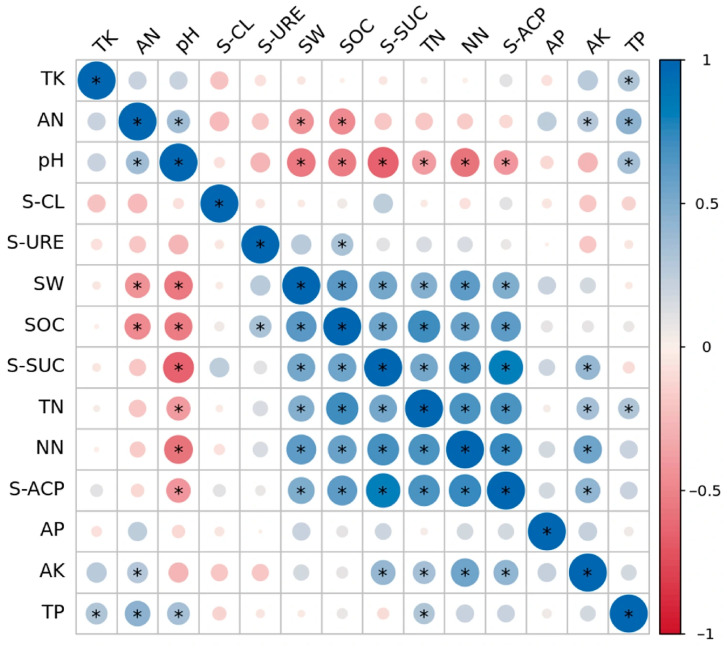
Correlation between soil physicochemical properties and soil enzyme activities. **Note**: The abbreviations used in the figure are as follows: AK, available potassium; AP, available phosphorus; TK, total potassium; TN, total nitrogen; NN, nitrate nitrogen; AN, ammonium nitrogen; SOC, soil organic carbon; SW, soil water content; S-ACP, acid phosphatase; S-SUC, sucrase; S-CL, cellulase; S-URE, urease. The color gradient and asterisks represent the strength and statistical significance of the correlations, respectively.

Correlation analysis revealed significant relationships among soil physicochemical properties and microbial indicators across different stand densities (Figure 7). TK and TP showed a significant positive correlation (r = 0.32, *p* < 0.05). AN was positively correlated with pH (r = 0.36) but negatively correlated with SW (r = −0.42) and SOC (r = −0.46). Soil pH demonstrated a positive correlation with TP (r = 0.35) but significant negative correlations with SOC (r = −0.53), TN (r = −0.39), SW (r = −0.56), S-ACP (r = −0.40), S-SUC (r = −0.66), and NN (r = −0.57). SW was positively correlated with SOC (r = 0.64), S-SUC (r = 0.53), TN (r = 0.47), NN (r = 0.61), and S-ACP (r = 0.49). SOC showed positive correlations with S-SUC (r = 0.54), TN (r = 0.71), NN (r = 0.56), and S-ACP (r = 0.61). S-SUC activity was positively correlated with TN (r = 0.52), NN (r = 0.70), S-ACP (r = 0.82), and AK (r = 0.41). TN was positively correlated with NN (r = 0.67), S-ACP (r = 0.68), AK (r = 0.35), and TP (r = 0.30). NN showed positive correlations with S-ACP (r = 0.72), AK (r = 0.54), and TP (r = 0.30). A significant positive correlation was also observed between S-ACP and AK (r = 0.42). All reported correlations were statistically significant (*p* < 0.05).

### 2.5. Main Environmental Factors Affecting Soil Microbial Community Structure

**Figure 8:**
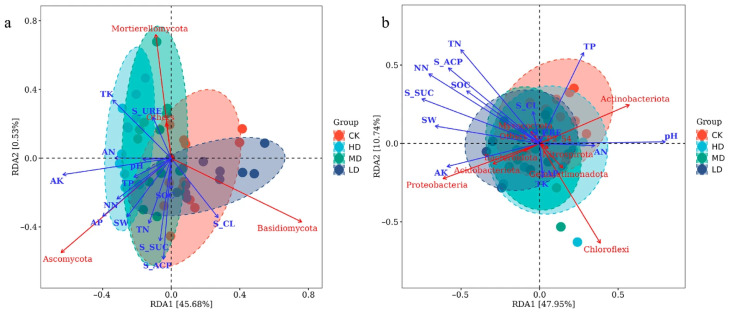
RDA ordination plot of soil fungal (**a**) and bacterial (**b**) communities and soil micro-environmental factors. **Note:** RDA, redundancy analysis. Environmental factors are represented by blue arrows with abbreviations: AK, available potassium; AP, available phosphorus; TK, total potassium; TN, total nitrogen; NN, nitrate nitrogen; AN, ammonium nitrogen; SOC, soil organic carbon; SW, soil water content; S-ACP, acid phosphatase; S-SUC, sucrase; S-CL, cellulase; S-URE, urease. Fungal and bacterial phyla are indicated by red labels. The length and direction of arrows indicate the strength and direction of the relationship between environmental variables and community composition.

Soil fungal communities were significantly influenced by soil micro-environmental factors under different stand retention densities (Figure 8a). RDA1 and RDA2 explained 45.68% and 0.53% of the variation, respectively, with AK, AP, TK, S-ACP, S-SUC, and S-CL identified as key environmental drivers. AK, AP, S-ACP, and NN showed strong positive effects on *Ascomycota*. In contrast, TK and S-URE exhibited strong positive effects on *Mortierellomycota* but strong negative effects on *Basidiomycota*; these trends were particularly evident in HD and MD stands. Conversely, S-CL demonstrated a strong positive effect on *Basidiomycota*, which was more pronounced in CK and LD plots.

Soil bacterial communities were also significantly affected by soil micro-environmental factors (Figure 8b). RDA1 and RDA2 accounted for 47.95% and 10.74% of the variation, respectively, with pH, TP, TN, NN, S-ACP, S-SUC, and SW identified as key influencing factors. pH and TP had strong positive effects on *Actinobacteriota*—particularly evident in CK and HD plots—but strong negative effects on *Proteobacteria* and *Acidobacteriota*. In contrast, AK and SW showed strong positive effects on *Proteobacteria* and *Acidobacteriota* but strong negative effects on *Actinobacteriota*. However, these patterns did not show clear differentiation across stand density levels.

## 3. Discussion

### 3.1. Effects of Stand Density on Soil Physicochemical Properties

The stand retention density induced variations in soil physicochemical properties revealed a clear depth-dependent response pattern, highlighting the critical role of forest management in regulating belowground biogeochemical cycles. The observed decline in soil pH with decreasing retention density aligns with previous reports that reduced canopy coverage accelerates litter decomposition and organic acid accumulation [9]. Notably, the “mid-density (MD)” treatment consistently exhibited optimal soil water retention and SOC content in 0–30 cm layers, suggesting moderate density balances microclimate regulation and nutrient input. This contrasts with high-density (HD) stands where intense root competition likely limited SOC sequestration, and low-density (LD) plots where diminished litter fall impaired carbon replenishment-a phenomenon well-documented in temperate plantations [10].

The stratification of nutrient dynamics across soil profiles reflects distinct ecological drivers. The significant reduction in TN and TP in surface layers (0–20 cm) under LD treatment may stem from enhanced leaching losses due to reduced vegetation interception, as demonstrated in Chinese larch plantations [11]. Conversely, the non-significant changes in deep layers (30–40 cm) across all treatments imply limited management impacts on geologic nutrient pools, consistent with findings from boreal forest ecosystems [12]. Nitrogen speciation responses further elucidate microbial mediation mechanisms. The progressive decline of NH_4_^+^-N and NO_3_^−^-N with retention density (HD > MD > LD) correlates with reduced nitrification rates under canopy opening, as observed in Mongolian oak forests [13]. The preservation of inorganic N pools in HD stands suggests that high density maintains sufficient shade to suppress nitrifier activity while allowing adequate through fall for ammonification-a delicate balance critical for preventing N leaching in managed forests.

The stratification of soil enzyme activities under varying retention densities demonstrates density-dependent functional adaptations in microbial-mediated biogeochemical processes. The elevated S-SUC and S-CL activities in LD and MD stands within 0–30 cm (Figure 2) likely reflect enhanced carbon substrate availability from accelerated litter decomposition, a phenomenon amplified by increased solar radiation penetration in thinned stands [14]. Contrastingly, S-URE activity peaked in HD stands within surface layers (0–20 cm), aligning with observations in *Pinus tabuliformis* Carrière plantations where canopy closure maintained higher nitrogen availability through reduced nitrification/denitrification losses [15]. This nitrogen conservation mechanism, however, appears compromised in LD treatments as evidenced by S-URE suppression, potentially exacerbating N limitation in intensively managed stands [16].

### 3.2. Effects of Stand Density on Soil Microbial Diversity and Community Composition

The density-driven shifts in fungal-to-bacterial dominance patterns unveil fundamental restructuring of belowground microbial functional guilds under retention densities. The progressive decline of *Ascomycota* and *Mortierellomycota* with decreasing retention density (Figure 3) parallels findings in *Pinus halepensis* Mill. forests, where canopy opening reduces cellulose-degrading specialists while favoring *Basidiomycota*-dominated wood decay fungi [17]. This functional transition-from litter decomposition (*Ascomycota*) to humus mineralization (*Basidiomycota*)-implies accelerated organic matter turnover in low-density stands, potentially explaining the observed SOC decline in surface layers. In bacterial communities, the density-dependent decrease in Actinobacteriota and concomitant rise in Acidobacteriota reflect carbon starvation strategies (Figure 3). Actinobacteria’s oligotrophic advantage in HD stands aligns with their high C-use efficiency under recalcitrant organic matter conditions [18]. Conversely, the *Acidobacteriota* dominance in LD treatments suggests metabolic adaptation to labile C inputs from understory vegetation-a pattern documented in North American hardwood forests [19]. The increased *Proteobacteria* further indicates r-strategist proliferation under resource-rich post-retention density environments [20].

Regarding the fungal community, the moderate density (MD) treatment exhibited the highest Chao1 and Shannon indices in the 10–20 cm soil layer. This aligns well with the ecological theory of “intermediate disturbance promoting diversity” [21]. Notably, the high density (HD) treatment showed the highest Simpson index in the topsoil layer (0–30 cm), indicating that its fungal community structure may be dominated by a few prevalent taxa (e.g., *Ascomycota*). This phenomenon is consistent with observations by Tedersoo et al. [22] in global forest ecosystems. The optimal performance of the MD treatment in the deeper soil layer (30–40 cm) likely stems from its creation of a more balanced resource allocation pattern, avoiding both intense competition under high density and resource limitation under low density. In contrast, the response of bacterial diversity to stand density was relatively muted, with the MD treatment consistently exhibiting optimal diversity indices across different soil layers. This response pattern likely reflects the higher functional redundancy [23] and more flexible environmental adaptation strategies [24] characteristic of bacterial communities.

The density-driven divergence in microbial community structures, as revealed by PCoA (Bray–Curtis distance), underscores fundamental differences in ecological resilience between fungi and bacteria. The higher explanatory power of bacterial PC1 (18.20% vs. fungal 12.70%) aligns with their metabolic plasticity under resource fluctuations-a phenomenon observed in tropical forest soils where bacterial communities rapidly adapt to altered carbon inputs [25]. Notably, the contrasting bacterial patterns-with CK/HD and MD/LD forming distinct clusters-reflect niche partitioning along pH gradients (6.38–6.89). *Acidobacteriota*’s dominance in LD soils (24.15%) correlates with their acidophilic traits, while *Actinobacteriota*’s prevalence in HD (21.81%) mirrors adaptations to near-neutral microsites, consistent with findings in managed Pinus sylvestris stands [26].

In this study, the fungal and bacterial communities exhibited differential response patterns to stand density manipulation (Figure 6), which reflects their distinct ecological strategies and functional roles. First, this divergence stems from their fundamental trophic niche differentiation. Fungi, particularly *Basidiomycota*, are the primary agents responsible for degrading complex organic matter such as lignin and cellulose [27]. The reduction in stand density increases light availability and litter lignification, directly creating a habitat favorable for *Basidiomycetes* (e.g., white-rot fungi) and leading to a relative decline in saprotrophic fungi like *Ascomycota*. In contrast, bacterial communities rely more heavily on readily decomposable root exudates and simple carbon sources [25]; their broader resource spectrum renders them less responsive to the selective pressure primarily driven by litter quality. Second, differences in life-history strategies and functional redundancy are crucial. Bacterial communities typically exhibit higher functional redundancy and are predominantly r-strategists. They can maintain stable overall metabolic functions through rapid population turnover [28]. Fungal communities, however, are mostly K-strategists. Their stable mycelial networks, once altered, recover more slowly. Consequently, their community structure shows more pronounced and persistent responses to long-term management measures, such as fixed-density silviculture. Finally, variation in key environmental drivers amplifies this disparity. Our study found that bacterial community structure was closely correlated with soil pH, which decreased with reducing stand density. This correlation directly drove the succession from *Actinobacteria* to *Acidobacteria*. Fungal communities were more sensitive to micro-physical habitats regulated by canopy structure (e.g., fluctuations in temperature and humidity) and to plant–fungal interactions (e.g., changes in host carbon allocation strategies to mycorrhizal fungi under different competitive pressures) [20]. The combined action of these multiple mechanisms ultimately results in fungal communities serving as more sensitive biological indicators of stand structural management history, while bacterial communities demonstrate greater functional resilience.

### 3.3. Relationship Between Soil Microbial Community Structure and Environmental Factors

This study revealed the significant influence of stand retention density on soil fungal community structure through RDA, identifying soil available nutrients (AK, AP) and enzyme activities (S-ACP, S-URE) as key environmental drivers of fungal phylum-level distribution. This finding aligns with the hypothesis of co-evolution between “soil chemical properties and microbial functional groups” in global forest ecosystems [17]. The first two RDA axes cumulatively explained 46.21% of the variation, significantly higher than similar studies in artificial forest of pine seedlings in Mu Us Desert in China (e.g., 20.52% in Hai et al. [29]), suggesting that fungal communities in temperate plantations exhibit heightened sensitivity to environmental factors. The analysis showed significant positive effects of AK and AP on *Ascomycota*, consistent with the ecological characteristics of this phylum as r-strategists. *Ascomycetes* typically possess a rich array of hydrolytic enzymes (e.g., chitinases, cellulases), enabling them to rapidly utilize simple carbon sources and available phosphorus [30]. In this study, the significantly higher AP content in high-density (HD) stands compared to the control (CK) likely promoted the enrichment of *Ascomycota* (Figure 1). These results support the theoretical framework of “phosphorus availability driving fungal community assembly” [31], particularly pronounced in intensively managed plantations. The positive association between TK and *Mortierellomycota* highlights the specific role of potassium cycling in fungal ecology *Mortierella* species have been confirmed to possess potassium-solubilizing capacity [32], and their dominance under high TK conditions may reflect a specialized nutrient acquisition strategy (Figure 1). Notably, the negative correlation between *Basidiomycota* and TK implies lower potassium requirements among lignin-degrading fungi (e.g., white-rot fungi), consistent with findings in temperate forests by Bödeker et al. [33]. The positive correlation between S-ACP and *Ascomycota* likely reflects a microbial adaptation strategy under phosphorus-limited conditions. When soil available phosphorus is insufficient, microbes secrete phosphatases to mineralize organic phosphorus [34]. This mechanism is corroborated by the significantly higher S-ACP activity in HD stands compared to other treatments (Figure 2). Conversely, the positive correlation between S-URE and *Mortierellomycota* may indicate this phylum’s role in nitrogen cycling; its capacity to hydrolyze urea for nitrogen acquisition has been confirmed in metagenomic studies [35]. Micro-environmental differentiation caused by varying retention densities significantly influenced fungal community assembly. The inverse relationship between *Mortierellomycota* and *Basidiomycota* in high-density stands (HD/MD) may originate from changes in carbon input quality driven by differences in canopy closure. Previous studies have demonstrated that high canopy closure promotes the accumulation of easily decomposable litter, favoring the growth of saprotrophic *Mortierellomycota* [36]. In contrast, low-density stands (LD) with ample sunlight favor the colonization of *Basidiomycota* due to litter with higher lignin content [37]. This interpretation is further supported by the positive correlation between S-CL and *Basidiomycota*, as this phylum includes important cellulose-degrading taxa.

This study revealed the significant influence of key environmental factors on soil bacterial community structure across different stand retention densities through RDA. The primary drivers identified were pH, TP, TN, NN, S-ACP, S-SUC, and SW. The first two RDA axes cumulatively explained 58.69% of the variation, indicating a strong filtering effect of the soil microenvironment on bacterial community composition. This result aligns with conclusions from global soil microbial biogeography research. pH emerged as one of the most critical factors influencing the bacterial community. The analysis showed a positive effect of pH on *Actinobacteriota* and negative effects on *Proteobacteria* and *Acidobacteriota*. This finding is consistent with the classical “soil pH-microbial diversity” theory [25], wherein actinobacteria are more adapted to neutral to slightly alkaline conditions, while *Acidobacteria* and certain *Proteobacteria* (e.g., *α-Proteobacteria*) prefer acidic environments [38]. The higher pH in this study may promote the growth of *Actinobacteria* due to their enhanced organic matter decomposition capacity, whereas under low pH, *Acidobacteria* may maintain competitive advantage through oligotrophic strategies [39]. The negative effects of TN and NN on *Chloroflexi* may reflect this phylum’s sensitivity to high nitrogen environments. *Chloroflexi* are typically found in oligotrophic soils and participate in decomposing recalcitrant organic matter [40]. High nitrogen input may inhibit their growth, consistent with findings from long-term fertilization experiments in agricultural soils [41]. Additionally, the negative correlations of S-ACP and S-SUC with *Chloroflexi* may indicate greater competitiveness of this phylum under conditions of low enzyme activity (i.e., low metabolic pressure), suggesting an oligotrophic growth strategy [42]. The positive effects of AK and SW on *Proteobacteria* and *Acidobacteriota* may be related to their metabolic flexibility. *Proteobacteria* (especially *γ-Proteobacteria*) encompass numerous strains capable of nitrogen fixation and diverse carbon metabolism [43], and higher SW may facilitate their dispersal and substrate utilization. In contrast, the negative response of *Actinobacteriota* to AK and SW may relate to their drought tolerance, as previous studies have shown *Actinobacteria* can maintain relatively high activity under dry conditions [44].

Unlike the fungal community, bacterial communities exhibited smaller differences among stand densities. This indicates that bacteria may possess higher functional redundancy [23]. This phenomenon supports the ecological theory that “microbial community assembly is governed primarily by environmental filtering rather than stochastic processes” [45]. Future research could integrate metagenomics to further dissect differences in bacterial functional genes across stand densities, enabling a more precise assessment of the impact of management practices on soil microbial functions.

Overall, the moderate-density (MD) treatment creates an optimal disturbance condition. (1) Regarding the litter-microbial feedback loop: MD creates a canopy structure that balances light penetration and litter input. This promotes the accumulation and efficient decomposition of medium-quality litter, facilitating a positive feedback loop. Enhanced labile carbon from litter fuels microbial activity, while the subsequent formation of microbial necro mass and stable byproducts directly contributes to the physical protection and accumulation of SOC. The functional shift towards Basidiomycota, as observed, is a key mechanism in this process, enhancing the breakdown of complex compounds and humification. (2) In terms of soil microclimate modulation: The moderately open canopy in MD stands acts as a buffer for the soil microclimate. It reduces evaporative water loss compared to LD stands while improving infiltration and reducing surface runoff compared to HD stands. This optimal regulation of soil moisture and temperature directly enhances water retention and creates more stable conditions for microbial activity and SOC stabilization, reducing the volatility of decomposition rates. (3) In the context of niche partitioning and resource heterogeneity: MD management increases resource heterogeneity by providing a diverse array of microhabitats and substrate types—from fresh root exudates to partially decomposed litter. This spatial and temporal variety in resources supports a broader spectrum of ecological niches. It allows for the coexistence of both oligotrophic (e.g., *Acidobacteriota*) and copiotrophic (e.g., *Proteobacteria*) strategists, as well as functionally distinct fungi (e.g., cellulose-degrading vs. lignin-degrading), thereby directly explaining the observed peak in microbial alpha-diversity. We will discuss how the MD environment fosters a tighter coupling of carbon, nitrogen, and phosphorus cycles. For instance, the improved water retention and SOC availability enhance microbial nutrient immobilization and reduce leaching losses. The simultaneous presence of diverse functional groups ensures that the products of one process (e.g., cellulose hydrolysis by fungi) become substrates for another (e.g., nitrogen mineralization by bacteria), leading to a more efficient and self-sustaining soil nutrient economy.

## 4. Materials and Methods

### 4.1. Study Site Description

The experimental area is located in the Shanggaotai Forest Farm, Daqing Mountain Management Area, Inner Mongolia Autonomous Region, China (115°51′–112°23′ E, 40°56′–41°15′ N). This region experiences a cold-temperate, arid to semi-arid continental monsoon climate, characterized by long sunshine hours (approximately 2735 annual average sunshine hours) and a frost-free period of about 120 days. The mean annual temperature is 6 °C, and the mean annual precipitation is approximately 337.5 mm. The study was conducted on a south-facing slope within the forest farm, with an average gradient of 15 degrees. The soil is classified as cinnamon soil, with a depth exceeding 90 cm and a slightly acidic pH. The *Larix principis-rupprechtii* plantation in this area was established over 40 years ago, with this species being a fast-growing timber tree. According to land use classification, the area is designated as plantation forest. The understory vegetation is predominantly composed of shrubs such as *Spiraea salicifolia* L., *Ostryopsis davidiana Decne*, and *Prunus sibirica* L., along with herbaceous species including *Stipa capillata* Linn., *Artemisia frigida Willd.*, and *Thymus mongolicus* (Ronniger) Ronniger.

### 4.2. Experimental Design and Soil Sampling

This study aims to compare the soil ecological effects under different management intensities. We established a control plot (CK) that has not undergone any thinning, representing the natural growth state and the highest stand density. Building upon this baseline, three target density gradients were created by implementing different intensities of commercial thinning: the High Retention Density (HD), Medium Retention Density (MD), and Low Retention Density (LD) treatments. It is important to note that even in the HD treatment, a portion of trees was removed through thinning; therefore, its stand density is lower than that of CK. This design allows CK to serve as the benchmark for evaluating the ecological effects of thinning measures, while HD, MD, and LD represent a continuum of management strategies from conservative to intensive. In the study area, four experimental sites were established, including three *Larix principis-rupprechtii* plantations with different stand retention densities (high (2077 trees ha^−1^), medium (1108 trees ha^−1^), and low (556 trees ha^−1^)) and one unharvested stand (CK (2800 trees ha^−1^)) as the control (Table 1). Soil samples were collected in August 2024. Within each forest stand, three 30 m × 30 m experimental plots were randomly established. All selected plots were free from livestock grazing and human disturbance, with a minimum inter-plot distance of 20 m. An adjacent unharvested stand was selected as a control for the three density treatments. Soil samples were collected from the 0–40 cm depth profile at 10 cm intervals using a sterile soil auger with a 5 cm diameter across all 12 plots.

To ensure soil sample homogeneity, samples were collected from four cardinal directions (east, south, west, and north) within each plot. Soil samples from each sampling point were thoroughly mixed to form a composite sample, ensuring representativeness. The composite samples were sieved through a 2 mm mesh to remove stones and plant residues. Subsequently, 1 kg of soil was placed into sterile zip-lock bags and transported to the laboratory in a −20 °C vehicle freezer. One portion of each sample was air-dried indoors and then oven-dried at 105 °C for 6 h to determine pH, organic matter, and total nitrogen content. Another portion was stored at 4 °C for subsequent analysis of ammonium nitrogen, nitrate nitrogen, and enzyme activities. Simultaneously, separate samples dedicated to microbial community analysis were preserved in liquid nitrogen during transport to maintain biological integrity. Upon arrival at the laboratory, these samples were promptly transferred to a −80 °C freezer for storage. To ensure analytical reliability, three replicate subsamples were prepared from each composite sample for subsequent soil microbial community analysis.

### 4.3. Determination of Soil Physicochemical Properties

Various analytical techniques were employed to assess soil chemical properties. Soil samples were mixed with distilled water at a 1:2.5 ratio, extracted for 30 min, and the suspension pH was measured using a pH meter (Mettler Toledo, Shanghai, China). Soil water content was determined by the oven-drying method. Soil organic carbon (SOC) content was determined via the potassium dichromate heating method. Total nitrogen (TN) was quantified using the Kjeldahl method, while total phosphorus (TP) was measured by sodium hydroxide digestion followed by molybdenum–antimony colorimetry. Available phosphorus (AP) was extracted with ammonium fluoride and hydrochloric acid and analyzed by molybdenum–antimony colorimetry. Available potassium (AK) was extracted with ammonium acetate and determined by atomic absorption spectrometry. Additionally, ammonium nitrogen (NH_4_^+^-N), nitrate nitrogen (NO_3_^−^-N), and soil enzyme activities were measured. Specific procedures and conditions followed established methodologies [46] (Epoch 2, BioTek, Instruments, Inc., Winooski, VT, USA).

This study determined the activities of four hydrolases associated with soil carbon, nitrogen, and phosphorus cycling: sucrase (S-SUC), cellulase (S-CL), urease (S-URE), and acid phosphatase (S-ACP). All enzyme activities were assayed using fresh soil samples. Each sample was analyzed in triplicate, with both soil-free and substrate-free controls included to correct for non-enzymatic reactions and soil background interference. The above enzyme activities were measured with a 96-well multifunctional enzyme analyser (SuPerMax 3100, Shanghai Shanpu Biotechnology Co., Ltd., Shanghai, China) according to the instructions of the Solarbio kit (Beijing Solarbio Science & Technology Co., Ltd., Beijing, China). S-SUC activity was measured using the 3,5-dinitrosalicylic acid (DNS) colorimetric method. One unit of enzyme activity (U) is defined as the amount of enzyme that catalyzes the production of 1 mg of reducing sugar from sucrose by 0.03 g of soil in 24 h. Activity was quantified based on the absorbance at 540 nm. S-CL activity was assayed by the DNS method using carboxymethyl cellulose as the substrate. One unit of activity (U) is defined as the amount of enzyme that produces 1 mg of glucose per gram of soil per day at 37 °C. Activity was determined based on the absorbance at 540 nm using 0.05 g of soil. S-URE activity was expressed as per μg NH_3_-N g^−1^ d^−1^ soil and determined according to the amount of fluorescence absorption at 630 nm by 0.25 g of soil sample; S-ACP activity was expressed as per nmol phenol g^−1^ d^−1^ soil and determined according to the amount of fluorescence absorption at 660 nm by 0.25 g soil sample.

### 4.4. DNA Extraction and Illumina Sequencing

Samples were retrieved from storage and promptly aliquoted (0.2–0.5 g) into centrifuge tubes containing extraction lysis buffer for homogenization using a Tissuelyser-48 multi-sample tissue grinder (Shanghai Jingxin, Shanghai, China) at 60 Hz. Total microbial genomic DNA was extracted from 0.5 g of fresh soil using the MagBeads FastDNA Kit for Soil (116564384, MP Biomedicals, Irvine, CA, USA) following the manufacturer’s protocols. DNA quality and concentration were evaluated by 1.0% agarose gel electrophoresis and a NanoDrop^®^ ND-2000 spectrophotometer (Thermo Scientific, Waltham, MA, USA), after which samples were stored at −80 °C for subsequent analysis. The bacterial 16 S rRNA gene and fungal 18 S rRNA gene were amplified with primer pairs 338F/806R [47,48] and ITS-1F/ITS-2R [49], respectively, on an ABI GeneAmp^®^ 9700 PCR thermocycler (Foster City, CA, USA). Each 20 μL PCR reaction contained 4 μL of 5× Fast Pfu buffer, 2 μL of 2.5 mM dNTPs, 0.8 μL of each primer (5 μM), 0.4 μL of Fast Pfu polymerase, and 10 ng of template DNA. The thermal cycling program consisted of initial denaturation at 95 °C for 3 min; 27 cycles of denaturation at 95 °C for 30 s, annealing at 55 °C for 30 s, and extension at 72 °C for 45 s; final extension at 72 °C for 10 min; and hold at 4 °C. All amplifications were performed in triplicate. Amplicons were purified from 2% agarose gels using the AxyPrep DNA Gel Extraction Kit (Axygen Biosciences, Union City, CA, USA) and quantified with a Quantus™ Fluorometer (Promega, Madison, WI, USA). Equimolar amounts of purified amplicons were pooled and subjected to paired-end sequencing on an Illumina MiSeq PE300 platform (Illumina, San Diego, CA, USA) following the standard protocols of Personal Biotechnology Co., Ltd. Shanghai, China (Shanghai, China).

### 4.5. Data Processing and Analysis

Bioinformatic analysis of bacterial and fungal FASTQ raw sequencing data was performed using the QIIME2 pipeline [50]. Quality filtering was conducted with fastp (v0.19.6) [51], followed by sequence merging using FLASH (v1.2.6) [52]. After quality control and merging, the DADA2 plugin [53] within QIIME2 [50] was employed to denoise the optimized sequences under default parameters. The resulting sequences from DADA2 processing are referred to as amplicon sequence variants (ASVs). All sequences annotated as chloroplasts or mitochondria were removed. To minimize the impact of sequencing depth on subsequent alpha and beta diversity analyses, all samples were rarefied to 20,000 sequences per sample. Following this normalization, the average sequencing coverage remained at 99.09%. Taxonomic annotation of ASVs was performed using a naive Bayes classifier against the SILVA 16 S rRNA gene database (v138) and the UNITE database (Release 8.0 https://unite.ut.ee/ (accessed on 10 October 2024)) for bacteria and fungi, respectively. Alpha diversity indices were calculated to assess microbial richness and diversity. The Chao1 and ACE indices were used to estimate ASV richness through different algorithms, while the Shannon index reflected both richness and evenness. The Simpson index indicated community dominance. All alpha diversity indices were computed through the Personal Biotechnology Cloud Platform (https://www.genescloud.cn/). Beta diversity, representing dissimilarities in microbial composition among communities, was visualized using principal coordinate analysis (PCoA) based on ASV profiles. Differences in soil physicochemical properties among stand densities were analyzed using SPSS Statistics (v25.0). One-way analysis of variance (ANOVA) with LSD post hoc testing (*p* < 0.05) was applied for multiple comparisons. Spearman’s rank correlation analysis was used to examine relationships between soil properties and enzyme activities. Microbial community composition was analyzed by calculating relative abundances at the phylum level. RDA (Canoco5 software, version 5.0) was employed to identify driving factors of soil properties on microbial community structure.

## 5. Conclusions

This study demonstrates that implementing moderate-density (MD) management in *Larix principis-rupprechtii* plantations is a key measure for optimizing belowground ecological processes and promoting forest sustainability. Its core advantage lies in creating an optimal disturbance window: the moderately open canopy not only directly enhances topsoil (0–30 cm) water-holding capacity and organic carbon sequestration but also, by regulating litter quality and the micro-environment, drives functional restructuring of the soil microbial community. Specifically, it promotes functional complementarity between *Basidiomycota* (responsible for *lignocellulose* degradation) and *Acidobacteriota* (adapted to oligotrophic conditions), thereby sustaining an efficient nutrient cycling system. This research provides direct evidence for precision forest management based on soil ecological health. Stand density serves not only as a tool for regulating tree growth but also as a lever for modulating soil biogeochemical cycles. Incorporating soil microbial community structure (e.g., *Ascomycota*/*Basidiomycota* ratio, *Actinobacteriota*/*Acidobacteriota* ratio) and key enzyme activities into management assessment frameworks can offer early, sensitive biological indicators for maintaining long-term productivity and soil fertility in plantations. The findings link stand structural management, soil ecological functions, and microbial mechanisms, deepening our understanding of sustainable forestry—which concerns not only timber yield but also the maintenance of a belowground ecosystem characterized by high diversity, high functional redundancy, and self-regulatory capacity. The soil health achieved under MD management forms the foundation for forest resilience to disturbances and long-term carbon neutrality and nutrient self-sustainability. To further elucidate the mechanisms and support practical application, future research should focus on long-term in situ monitoring to clarify the lasting ecological effects of MD management and its buffering capacity against climate change and integrating metagenomics and metabolomics to quantitatively identify the functional genes and metabolic pathways of key microbial taxa (e.g., *Basidiomycota*, *Acidobacteriota*) involved in carbon, nitrogen, and phosphorus cycling. This will provide predictive tools for determining optimal density strategies across diverse site conditions.

## Figures and Tables

**Table 1:** Basic information of the survey sample site.

Stand Density	Longitude	Latitude	TH (m)	DBH (cm)	UBH (m)	CB (m)
556 trees ha^−1^	112°04′29″ E	41°05′11″ N	15.6 ± 0.35 a	22.1 ± 0.48 a	4.2 ± 0.52 b	4.5 ± 0.31 a
1108 trees ha^−1^	112°03′48″ E	41°05′36″ N	15.2 ± 0.88 a	19.9 ± 0.17 a	4.4 ± 0.15 b	4.69 ± 0.81 a
2077 trees ha^−1^	112°04′05″ E	41°05′23″ N	14.8 ± 0.15 a	16.04 ± 0.04 a	4.9 ± 0.71 b	3.26 ± 0.37 a
2800 trees ha^−1^	112°03′29″ E	41°05′33″ N	14.5 ± 0.12 a	15.75 ± 0.44 a	5.3 ± 0.15 a	2.73 ± 0.64 a

Note: Different lowercase letters indicate significant differences in forest density between stands (*p* < 0.05). TH: tree height; DBH: diameter at breast height; UBH: under branch height CB: crown breadth.

## Data Availability

The original contributions presented in this study are included in the article. Further inquiries can be directed to the corresponding author.
